# Advances in drug delivery to atherosclerosis: Investigating the efficiency of different nanomaterials employed for different type of drugs

**DOI:** 10.1016/j.mtbio.2023.100767

**Published:** 2023-08-07

**Authors:** Binura Perera, Yuao Wu, Nam-Trung Nguyen, Hang Thu Ta

**Affiliations:** a School of Environment and Science, Griffith University, Nathan, Queensland, 4111, Australia; b Queensland Micro-Nanotechnology Centre, Griffith University, Nathan, Queensland, 4111, Australia

**Keywords:** Atherosclerosis, Nanomaterial, Drug delivery, Therapeutics, Plaques

## Abstract

Atherosclerosis is the build-up of fatty deposits in the arteries, which is the main underlying cause of cardiovascular diseases and the leading cause of global morbidity and mortality. Current pharmaceutical treatment options are unable to effectively treat the plaque in the later stages of the disease. Instead, they are aimed at resolving the risk factors. Nanomaterials and nanoparticle-mediated therapies have become increasingly popular for the treatment of atherosclerosis due to their targeted and controlled release of therapeutics. In this review, we discuss different types of therapeutics used to treat this disease and focus on the different nanomaterial strategies employed for the delivery of these drugs, enabling the effective and efficient resolution of the atherosclerotic plaque. The ideal nanomaterial strategy for each drug type (e.g. statins, nucleic acids, small molecule drugs, peptides) will be comprehensively discussed.

## Graphical abstract

**Figure:**
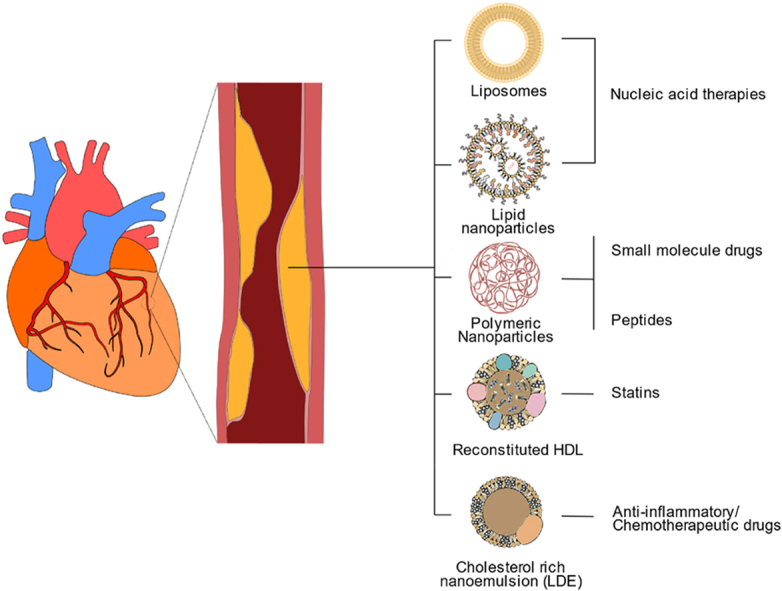

## 1 Introduction

Cardiovascular diseases (CVDs) are the leading cause of mortality worldwide, responsible for 19 million deaths in 2020 [1], making it the principal cause of death in middle- and high-income countries for over a decade [2]. Atherosclerosis is one of the major causes of CVDs due to the formation of atherosclerotic plaques that obstruct blood flow to the organs [3]. Clinical manifestations of atherosclerosis include ischaemic heart disease (IHD), stroke and peripheral arterial disease [4].

‘Atherosclerosis’ originates from the Greek words athero (gruel or paste), describing the appearance of plaque and sclerosis, which means the hardening of body tissue [5]. As a chronic inflammatory disease, atherosclerosis occurs in the arterial walls due to the imbalance of lipids in the bloodstream. When the concentrations of low-density lipoprotein cholesterol (LDL-c) are higher than the physiological levels, the LDL-c begin to passively diffuse from the arterial lumen to the tunica intima via endothelial junctions [3]. Subsequently, endothelium activation promotes the migration of monocytes for the removal of LDL. During the differentiation of monocytes into macrophages, reactive oxygen species (ROS) are produced, which oxidise LDL. The oxidised LDL (ox-LDL) is phagocytosed by the macrophages forming foam cells which recruit vascular smooth muscle cells (VSMC) from the tunica media [3,5]. By secreting matrix metalloproteinases 2 and 9, the migrated VSMCs produce collagen and elastin which aid in the formation of a fibrous cap on the plaque [3,5]. However, due to the inefficient clearance of the necrotic foam cells, VMSC and ox-LDL from the plaque, the inflammation persists and causes further endothelial dysfunction. This chronic inflammatory process occurs over time until either the plaque becomes large enough to disrupt the arterial lumen, impeding blood flow to the tissue, leading to ischemia (stable plaque), or the plaque ruptures and forms a thrombus (unstable plaque) [[3], [4], [5], [6], [7], [8], [9], [10], [11]]. Furthermore, areas of disturbed flow (d-flow) such as arterial bifurcations and areas of vessel curvatures are more prone to the development of lesions due to the decrease in mechanical forces and endothelial shear stress [12,13].

The primary treatment in early atherosclerosis aims to manage the major risk factors such as hypercholesterolemia, hypertension, and hyperglycaemia [14,15]. Dietary changes, regular exercise, and smoking cessation are all key to preventing and managing atherosclerosis [5,16]. The benefits of exercise on CVDs are further confirmed by the HUNT study [17], which is one of the largest health studies performed, concluded that having a higher physical activity is associated with a reduced risk of premature CVD in both healthy and individuals with CVD risk factors [17]. There are other unmodifiable risk factors such as age, gender, and genetics of the individual [14,15]. Since atherosclerosis is a multifactorial disease triggered by different risk factors, there are several methods, both clinical and under development, for treating this disease.

Medications prescribed encompass a range of drugs targeting hypercholesterolemic, hypertensive, and hypoglycaemic conditions as well antiplatelet drugs [[3], [4], [5],[14], [15], [16],^18^]. Anti-inflammatory drugs have also been studied for the treatment of atherosclerosis in clinical trials (including CANTOS for Canakinumab [19], CIRT for methotrexate [20], LoDoCo [21] and COLCOT [22] for colchicine). However, further evaluation is necessary to determine the therapeutic effects of these approaches [23,24]. All these drugs are aimed at preventing plaque progression and alleviating the symptoms associated with the disease, typically necessitating a regimen involving a combination of different drug categories. These drugs have been clinically proven to be safe to use and are almost exclusively delivered orally. Nonetheless, side effects of these medications exist. For instance, the employment of statins for the management of hypercholesterolemia can lead to intolerance manifested as statin-associated muscle symptoms (SAMSs). Furthermore, the use of statins has been linked with type 2 diabetes mellitus among other complications [25].

Despite being the most common route of administration due to its ease and high patient compliance, oral delivery of drugs faces numerous obstacles before reaching the target site. One main barrier is the first pass effect, where the drugs have a reduced concentration when they are systemically absorbed. Furthermore, due to a lack of targeting, the chances of presenting off-target effects are greater. Intravenous (IV) drug delivery on the other hand bypasses this as the drug is directly introduced to the systemic circulation, however, IV administration has its drawbacks such as infection, and damage to the veins and the injection site [26]. Regardless of the route of administration nanoparticle mediated drug delivery systems follow, they can be modified with coatings and binding ligands to protect themselves from the harsh gut environment, liver uptake and for the targeted delivery to the disease site, preventing off-target effects [27].

Surgical intervention is reserved for advanced cases of atherosclerosis, however, have their own risks. Invasive procedures, such as coronary artery bypass grafting and carotid endarterectomy, are associated with inherent risks during both the peri-operative and post-operative phases [28,29]. Non-surgical procedures such as percutaneous coronary intervention (PCI) contain several risks such as restenosis and in-stent restenosis [30]. Furthermore, a recent study showed that revascularisation of the artery by PCI and receiving optimal medical care (individually adjusted pharmaceutical treatments and device therapy for heart failure) did not improve the life expectancy of patients having severe left ventricular systolic dysfunction when compared with patients receiving only optimal medical care [31]. This trial (REVIVED-BCIS2) demonstrated comparable left ventricular ejection fraction between the two groups at 6 and 12 months. However, the quality-of-life scores favoured the PCI group for the first two years of the study [31].

**Fig. 1:**
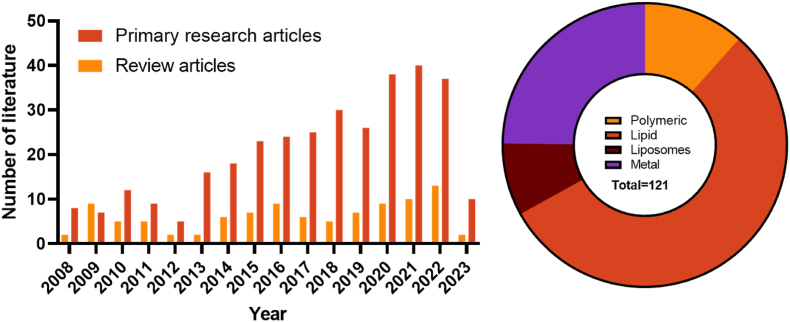
Number of journal articles and reviews published on the applications of nanomaterials for atherosclerosis (left), with the most popular nanomaterial types used for atherosclerosis (right) (Data from the NCBI [52]).

**Fig. 2:**
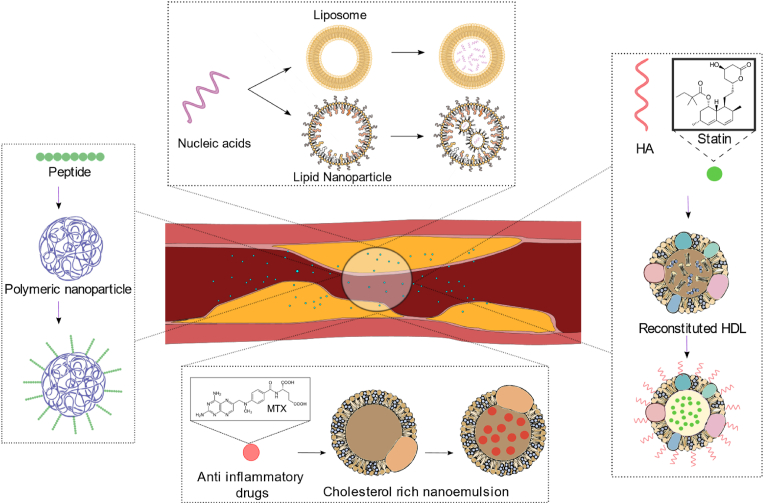
Different nanomaterial strategies employed to deliver therapeutic agents to the atherosclerotic plaque. (HA - Hyaluronic acid; MTX - methotrexate).

There have been reviews discussing nanomaterials for the treatment of cardiovascular diseases [[32], [33], [34]], which cover a broad range of treatments for coronary artery disease and ischemic heart disease. Several reviews have also been conducted on nanomaterials for the diagnosis and treatment of atherosclerosis [[35], [36], [37], [38], [39], [40], [41], [42], [43]], discussing the different types of nanomaterials and the targeting principles used to target specific sites of the atherosclerotic plaque to prevent plaque progression [[44], [45], [46], [47], [48], [49], [50], [51]]. Fig. 1 shows the increase in literature related to nanomaterials used in atherosclerosis over the past 15 years with the most popular nanomaterials used for this application in the last 10 years [52]. However, there hasn't been a review focusing on the drugs used to treat the plaque and the nanomaterials employed to deliver each type of drug. Therefore, in this review, we focus on emerging treatment options and discuss different nanomaterial strategies developed to efficiently deliver different types of therapeutics to atherosclerotic plaques (Fig. 2). The ideal nanomaterial strategy for each drug type (e.g. statins, nucleic acids, other small molecule drugs and peptides) will be determined by assessing the loading efficiency, loading capacity as well as treatment efficacy of the delivery system. This review is however limited by the information presented in the literature on the nanomaterials, which does not include factors such as the degradation profile of the nanomaterials, stability, toxicity, and scalability for clinical translation. The therapeutic efficacy of the nanoparticle treatment was assessed on the limited information reported in the literature which does not always discuss the delivery efficiency of the injected dose to the atherosclerotic plaque.

## 2 Nanomaterial strategies for targeted therapy

The use of nanomaterials for the targeted delivery of drugs has been a growing interest as it allows for materials in the nanoscale (1–100 nm) to serve as diagnostic tools or deliver drugs to specific target sites in a controlled manner [53,54]. Nanoparticles (NPs) can be broadly classified into two types: organic and inorganic NPs. Lipid NPs, micelles, liposomes, polymeric NPs, and carbon nanotubes (CNTs) are some of the examples categorized under organic NPs [54]. Examples of inorganic NPs include metal oxide NPs and mesoporous silica NPs [54].

**Fig. 3:**
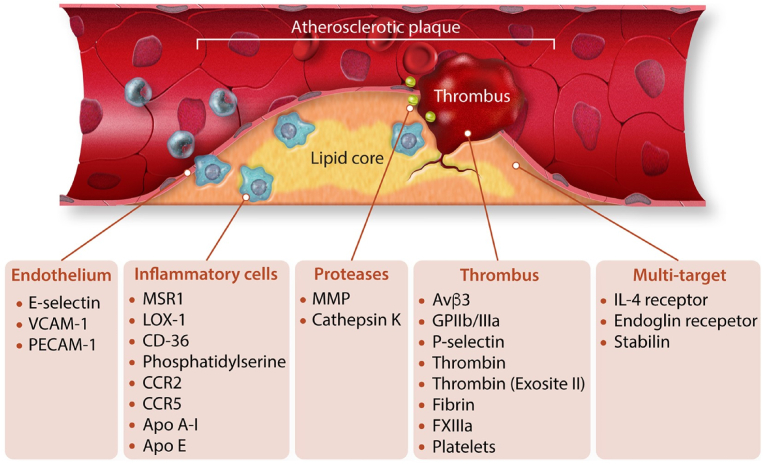
Different types of therapeutic targets for site specific delivery of nanoparticles in the atherosclerotic plaque [57].

The two types of targeting mechanisms associated with nanoparticle-mediated drug delivery are passive and active targeting. Passive targeting is nonspecific and relies on the enhanced permeability and retention (EPR) effect where the NPs accumulate in the region of the arterial wall in the atherosclerotic lesion due to the increase in vascular permeability. Active targeting relies on the receptor mediated uptake of nanoparticles into the diseased area [53,55], made possible due to the ability of surface modifications on nanoparticles [56]. There are several target sites for the site-specific delivery of NPs in atherosclerosis (Fig. 3) such as cell adhesion molecules, inflammatory cells, proteases and the extracellular matrix which are extensively reviewed by Zia et al., 2020 and Nasr and Huang, 2021 [57,58].

The present review discusses the different types of therapeutic agents used to treat atherosclerosis and the strategies employed for nanoparticle mediated drug delivery (Fig. 2). As the solubility of individual drugs are different, the strategies needed to facilitate the delivery of hydrophilic and hydrophobic drugs differ. Several issues faced by hydrophilic drugs include their poor pharmacokinetics caused by their inability to effectively pass-through lipid membranes, rapid metabolism, and renal clearance [59,60]. Hydrophobic drugs undergo suboptimal delivery of the drugs due to poor water solubility leading to low bioavailability [61]. All these limitations can be addressed by nanoparticle mediated drug delivery. Another advantage of nanoparticle mediated drug delivery is the co-delivery of drugs. Co-delivery of drugs is when a combination of two or more drugs is loaded to a delivery vector to increase the efficacy and safety of the therapy [62]. These drugs are aimed to work synergistically and reduce side effects. This strategy is increasingly popular in cancer therapy with several reviews published on the topic [[63], [64], [65]]. There are several studies on the co-delivery of different drugs for atherosclerosis as well [[66], [67], [68], [69], [70], [71], [72], [73], [74], [75]], however, this review focusses on the monotherapy of the different drug types and the nanomaterials used.

As different drug types have different mechanisms of action to prevent the progression of atherosclerosis, the following sections in this review will discuss how each drug type works to reduce plaque progression followed by the optimal nanomaterial which could be used to enhance the therapeutic efficacy.

### 2.1 Strategies for targeted delivery of statins

Statins have been used in the primary and secondary prevention of heart disease since its discovery in the 1970s by Akira Endo [76]. Statins target hepatocytes, inhibiting the rate-limiting enzyme in the metabolic pathway of cholesterol biosynthesis, 3-hydroxyl-3-methylglutaryl coenzyme A (HMG-CoA) reductase, which converts HMG-CoA to mevalonate (a substrate for cholesterol synthesis). The reduction of intracellular cholesterol causes the upregulation of LDL receptors in hepatic cells. Therefore, statins lower serum LDL levels in a non-linear, dose-dependent fashion. The mevalonate pathway, which is responsible for the biosynthesis of cholesterol, is also responsible for the synthesis of non-steroid isoprenoids [[77], [78], [79]]. These isoprenoids are the precursors used to synthesise dolichols which is important in glycoprotein synthesis [[77], [78], [79], [80]]; ubiquinone which is important for energy production in myocytes [81], and in the process of prenylation which is important in the activation of proteins for cell signalling pathways [82]. The interference of the mevalonate pathway by statins offer ‘pleiotropic’ effects such as increased endothelial nitric oxide synthase expression, increased fibrinolytic activity, pro-angiogenic effects, and immunomodulatory and anti-inflammatory effects, providing vascular protection [79]. However, side effects such as myalgias are frequent among statin users where statin induced muscle symptoms (SAMs) are the main reason for statin discontinuation [25]. Less common side effects include the onset of type 2 diabetes mellitus, neurological and neurocognitive effects, hepatotoxicity, and renal toxicity [78]. A meta-analysis of the use of statins and its effect on the plaque composition showed that statins lead to plaque healing and stabilisation but do not indicate plaque regression [83]. To achieve these local effects of statins, several studies have been conducted utilising nanoparticle carriers to deliver statins to atherosclerotic plaques (Table 1).

**Table 1:** Nanoparticle strategies for the delivery of statins to the atherosclerotic plaque.

Therapeutic Agent	Type of nanoparticle	Key nanoparticle materials	Size/Charge	Loading capacity (LC) and loading efficiency (LE)	Route of delivery	Injected dose	Animal model	Targeting strategy	Key Findings	Ref
**Pitavastatin** (lipophilic)	Polymeric Nanoparticle	PLGA	159 nm/-4mV	LC = 12.0% (w/v)	IV injection	Weekly injection of 0.012 mg of pitavastatin for 4 weeks	ApoE−/− mice	N/A NP uptake by macrophages	34% decreased plaque area in treatment compared to control	[84]
**Atorvastatin** (lipophilic)	Polymeric Nanoparticle	amphiphilic oxidation sensitive chitosan oligosaccharide	∼207 nm/∼-26 mV	LE = 48.3% LC = 5.1%	IV injection	5 x 10^7^ cells with NP internalised at an efficiency of ∼14.7%, injected weekly for 9 weeks	ApoE−/− mice	Macrophage membrane coating to prevent the clearance of the NPs from RES.	Decreased plaque area of ∼8% in MM-AT-NP compared to the saline group which had ∼20% plaque area of the total aorta tissue area	[85]
	Core/shell Nanoparticle	**Core:** hydrophobic statin aggregate **Shell:** Hyaluronic acid	122 nm/−35 mV (water) 135nm/−17 mV (PBS)	LC = 35%	IV injection	8.5 mg atorvastatin per kg animal body weight administered every other day, total 4 doses	ApoE−/− mice	Hylaluronic acid (HA) targeting the CD44 receptors in atherosclerotic plaque	Total plaque area in treatment group was 69% less than the control group	[86]
**Simvastatin** (lipophilic)	Liposome	DSPC DSPE-PEG2000	208.90 ± 3.99nm/−20 mV	LE = 93.57 ± 1.28% LC = 43.39 ± 7.6%	IV injection	10 mg/kg of simvastatin, 1 mg/kg epigallocatechin gallate (EGCG) twice a week for 3 weeks	ApoE−/− mice	N/A	SE-LNP treatment significantly reduced total aortic plaque area (<10%) compared to model (∼28%)	[87]
	Reconstituted HDL nanoparticle	rHDL	25–30 nm	LE = N/A LC = 11.6% (w/v)	IV injection	15 mg/kg of statin in rHDL bi-weekly for 12 weeks	ApoE−/− mice	N/A	31% decrease in total plaque area compared to the model group	[88]
		rHDL	138.2 ± 2.7 nm/-28.38 ± 0.52 mV	LE = 90.64 ± 0.43% LC = 5.03 ± 0.32%	IV injection	0.4 mg/kg every other day for 8 weeks	Male NZ white rabbits	Hylaluronic acid (HA) targeting the CD44 receptors in atherosclerotic plaque	Lesion positive staining showed a 10.9% lesion area of the treatment group compared to the 86.8% lesion area present in the model group	[89]
	Core/shell nanoparticle	**Core:** cyclodextrin/statin complex **Shell:** phospholipid	104 ± 13 nm/−20 ± 0.8 mV	N/A	IV injection	15 mg/kg of statin and 100 mg/kg of cyclodextrin 2,5, 8 and 11 days after LCA ligation	ApoE−/− mice	N/A	∼76% reduction in total plaque area in the treatment group compared to the control group	[90]
	Polymeric Nanoparticle	amphiphilic diblock copolymer (PEG-Ptyr-EO)	131.5 ± 6.4 nm/38.4 ± 9.7 mV	LE = 84.3% LC = 7.3%	IV injection	30 mg/kg of statin once a week for 4 weeks	ApoE−/− mice	Hylaluronic acid (HA) targeting the CD44 receptors	∼50% decrease in plaque area in the treatment group compared to control group	[91]
**Lovastatin** (lipophilic)	Reconstituted HDL nanoparticle	rHDL	152.9 ± 2.4 nm/-25.66 ± 0.65 mV	LE = 90.21 ± 0.50% LC = 4.34 ± 0.59%	IV injection	0.4 mg/kg, every other day for 8 weeks	Male NZ white rabbits	Hylaluronic acid (HA) targeting the CD44 receptors	Lesion positive staining showed an approximate 82% decrease in lesion area in treatment group compared to the model group	[92]
**Rosuvastatin (RSV)** (hydrophilic)	Mesoporous silica nanoparticles	Silica	137.5 nm /−16.3 mV	LE = 48.15 ± 0.95% LC = 8.78 ± 0.16%	IV injection	10 mg/kg for statin and 1.25 mg/kg for anti-CD9, injected every 2 days for a total of 8 doses	ApoE−/− mice	Anti-CD9 antibody targeting CD9 present in macrophages in the plaque environment	10.96% and 23.99% decrease of blood cholesterol level compared to free drug and control (PBS) respectively Treatment group had a lower plaque burden (7.5%) compared to the model group (13.3%)	[93]

The most popular nanomaterials used to encapsulate lipophilic statins (i.e. pitavastatin, atorvastatin, simvastatin, lovastatin) are polymeric nanoparticles and reconstituted HDL nanoparticles (rHDL). While most nanoparticles could encapsulate statins with loading efficiency (LE) in the range of 80–90%, the loading capacity (LC) was much lower, within 4–15%. Due to their hydrophobic nature, loading lipophilic statins into rHDL was expected to yield good loading efficiencies due to rHDL hydrophobic core [94]. However, it was not always the case. Duivenvoorden et al. [88] were able to encapsulate simvastatin in rHDL nanoparticles with a LC of 11.6%, however Zhang et al. [89] could only manage to get 5% LC. Lovastatin was loaded in rHDL nanoparticles at a lower capacity (4.34%). On the other hand, PLGA NPs could load pitavastatin with an impressive LC of 12% [84]. Amphiphillic polymers such as oxidation sensitive chitosan oligosaccharide [85] and amphiphilic diblock copolymer poly (ethylene glycol)-poly (tyrosine-ethyl oxalyl) (PEG-Ptyr-EO) [91] encapsulated atorvastatin and simvastatin with LCs of 5.1% and 7.3%, respectively. The use of Ptyr-EO block here was interesting as it was able to react with the hydrogen peroxide (H_2_O_2_) in the plaque area, reducing the H_2_O_2_ concentration and releasing the statin to exert its local anti-inflammatory action [91].

Liposomes were also employed to load simvastatin in a study by Wan et al. [87] and offered much greater loading capacity (43%) than polymeric and rHDL nanomaterials. Core/shell NPs comprising the hydrophobic atorvastatin aggregate as a core and hyaluronic acid (HA - a naturally existing polysaccharide) as a shell also provided great LC at 35% [86]. Core/shell structure was also employed to load simvastatin but the LC was not reported [90]. In this study, Kim et al. designed a core shell structure composed of an inclusion complex of cyclodextrin and simvastatin as a core and a lipid outer layer as a shell. This strategy used the ability of the cyclodextrin to bind to cholesterol crystals as a cargo switching nanoparticle. Due to the higher affinity of cyclodextrin to cholesterol crystals than the statin, the ‘cargo switching’ takes place in cholesterol rich environments, where the statins are released, and the cyclodextrin particles scavenge the cholesterol [90].

Inorganic nanoparticles have been used to encapsulate hydrophilic statins. HA coated mesoporous silica nanoparticles modified with CD9 antibodies for targeting were used to encapsulate rosuvastatin with a LE of 48.15 ± 0.95% and a LC of 8.78% [95]. This study aimed at utilising the anti-senescence effect of rosuvastatin and anti-CD9 antibody to alleviate the progression of atherosclerosis in mouse models. CD9 is a surface protein overexpressed in inflammatory macrophages and can promote cellular senescence. Anti-CD9 antibody was employed not only as a targeting ligand to target the plaque but also as a therapeutic agent to limit the progression of cellular senescence. In addition to the anti-inflammatory action of rosuvastatin, another pleiotropic effect of this statin is mediating cellular senescence by modulating the telomere maintenance system [95]. The HA coating acts as a stimuli-based response to the HAases which would allow for the release of the anti-CD9 antibodies and the rosuvastatin in the NPs. This is an example of a drug co-delivery strategy where both the antibody and rosuvastatin work synergistically to improve the therapeutic efficacy.

**Fig. 4:**
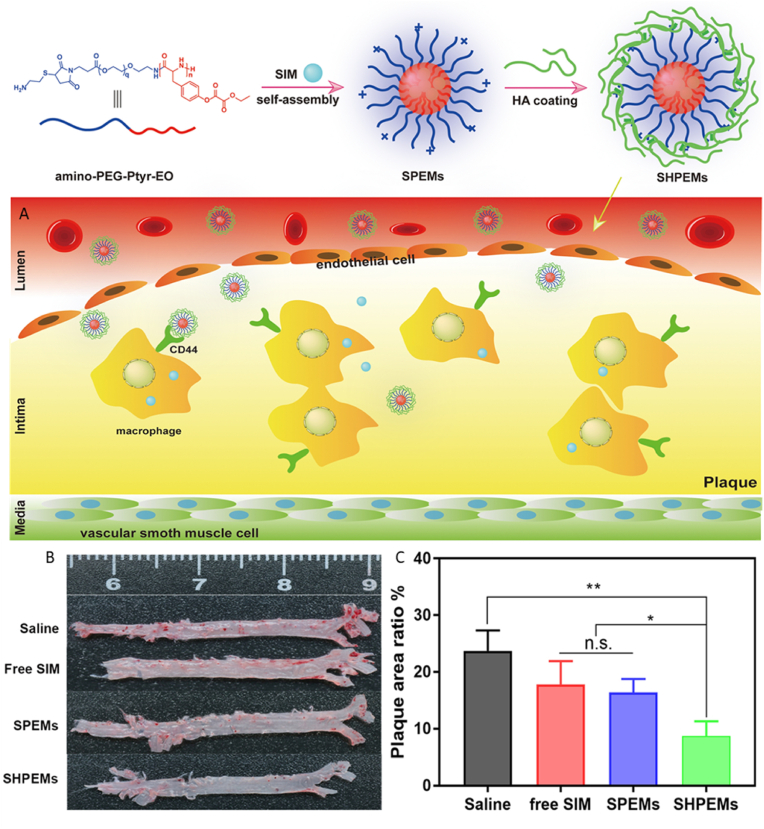
Nanomaterial strategy using HA coated polymeric nanoparticles to deliver statins to the atherosclerotic plaque. **(A)** Preparation of drug loaded nanoparticle and delivery into disease site. **(B)** Oil Red O (ORO) stained aortas. **(C)** Ratio of plaque area [91]. HA, Hyaluronic acid; SPEM, SIM-loaded amino-PEG-Ptyr-EO micelles; SPHEM, SIM-loaded HA-coated amino-PEG-Ptyr-EO micelles. Figures reproduced with permission from Mu et al., 2020. (For interpretation of the references to color in this figure legend, the reader is referred to the Web version of this article.)

Most of the NPs had sizes in the range of 100–200 nm and had negative zeta potentials. They were administered into atherosclerosis animal models (i.e. ApoE−/− mice, male NZ white rabbit) via IV injection. Most studies employed HA as targeting ligand for the CD44 receptor present on the surface of injured endothelium to target the atherosclerotic plaques [89,91,86,92]. HA coating was also shown reduced liver uptake of the NPs [89]. Other advantages of HA are that it creates a hydrophilic matrix preventing leakage of the hydrophobic drugs, and HA is broken down by hyaluronidase (HAase) which is an enzyme abundant in the plaque [89,92]. Fig. 4 shows an example of using HA coating on polymeric micelles. Macrophage coating was also employed in one study by Gao et al. to prevent the clearance of the NPs from the reticuloendothelial system (RES), which improved targeting to the lesion site and scavenged for pro-inflammatory cytokines [85]. While most studies reported the targeted delivery of NPs to the plaques, it is impossible to evaluate and compare the targeting efficiency of different NPs as these studies did not report this information.

Statins were loaded into the NPs alone or together with other therapeutics such as cyclodextrin (mentioned above) [90] or the antioxidant epigallocatechin gallate (EGCG) [87] to achieved synergistic effects. Wan et al. [87] used EGCG as it possesses strong antioxidant activity as well as promotes the polarization of M2 macrophages, in addition to the simvastatin to exert its anti-atherosclerotic activity. This particle shows slow release of its contents with only 62% release in PBS in 35 days. Higher release of 80% was seen in H_2_O_2_ environment. Other in vitro release profiles of polymeric NPs loaded with simvastatin showed 30.6% of cumulative drug release in 72 h which was increased to 54.3% with the addition of H_2_O_2_ [91]. Similarly, atorvastatin loaded polymeric NPs showed ∼20% of the drug release over 24 h which increased to ∼80% in the presence of H_2_O_2_ [85]. The in vitro release of rHDL was slower compared to polymeric NPs for simvastatin where a cumulative release of ∼20% was observed after 72 h [89], with another statin (lovastatin) showing 40% release by rHDL in the same time period [92].

All studies reported reduced plaque areas when atherosclerotic animals were treated with statin-loaded NPs. However, it is challenging to evaluate and compare the therapeutic efficiency delivered by different NPs from these different studies. This is because the treatment doses and the NP administering frequency were varied amongst the studies. In addition, some studies employed other therapeutic agents in addition to statins to achieve improved outcomes. Most studies reported the reduction of plaque areas by 10–80% compared to the control non-treated ones. In these studies, 8–15 mg statin/kg animal was employed every other day, twice a week, bi-weekly or weekly. Interestingly, rHDL nanoparticles loaded with simvastatin [89] and lovastatin [92] were able to reduce nearly 70% and 82% of plaque areas, respectively, when only a small dose of 0.4 mg statin/kg of rabbit was administered every other day for 8 weeks. These results may indicate the high efficiency of using rHDL NPs to deliver statins. However, rabbits were not employed in the studies using other types of nanomaterials, so it is not absolutely appropriate to make a solid conclusion.

Both polymeric [85,91,86,90] and reconstituted HDL nanoparticles [88,89,92] have shown greater efficacy in nanoparticle mediated delivery compared to the oral delivery of the drug. To ensure the statins have an enhanced circulation time in the plaque area, an optimal strategy involves utilising reconstituted HDL nanoparticles coated with HA. As discussed previously, the addition of HA coating further improves the therapeutic efficacy as HA ensures specific delivery to the inflammation site, making it a useful targeting ligand for any nanoparticle-mediated delivery system for atherosclerosis [96]. Furthermore, it prevents leakage of hydrophobic drugs and has a stimuli response release of the drugs in the plaque due to the degradation of the coating by HAase.

### 2.2 Strategies for targeted delivery of nucleic acids

Nucleic acid-based therapies such as RNA based antisense oligonucleotides (ASOs) and small interfering RNA (siRNA) are one of the recent potential treatments for atherosclerosis [97]. These therapies act by inhibiting the function of the target gene. Examples include the prevention of leukocyte recruitment by either downregulating adhesion molecule expression or preventing monocyte adhesion to endothelial cells [[98], [99], [100], [101], [102]], inhibiting pro atherogenic micro-RNAs (miRNAs) [103,104], promoting reverse cholesterol transport [105], and activating autophagy to prevent foam cell formation [106]. Despite having potential in the treatment of atherosclerosis, the drawbacks in the *in vivo* delivery of these therapies are degradation by RNAses and rapid renal clearance.

The solubility of nucleic acids plays an important role in understanding the optimal strategy for the delivery of RNA therapies. As with all nucleic acids, RNA has a negatively charged phosphate group in its sugar-phosphate backbone making them polar molecules, thereby hydrophilic. Table 2 summarises the different nanoparticle strategies employed to deliver nucleic acid therapies to the atherosclerotic plaque.

**Table 2:** Nanoparticle strategies for the delivery of nucleic acid therapies to the atherosclerotic plaque.

Therapeutic Agent	Nanoparticle	Key nanoparticle materials	Size/Charge	Loading capacity (LC) and loading efficiency (LE)	Route of delivery	Injected dose	Model	Targeting strategy	Key Findings	Reference
VCAM1, ICAM1 and 2, E− and P-selectin siRNA to silence the gene expression of the cell adhesion molecules	Polymeric Nanoparticle	7C1 compound synthesised by reacting C15 epoxide-terminated lipids with PEI600	45 ± 16 nm	N/A	IV injection	3 mg/kg of siRNA in 10 μL/g body weight. Injected days 1 and followed by weekly injections	ApoE−/− mice	N/A	Decrease in leukocyte recruitment and plaque inflammation Reduction in necrotic core and lesion size Increase in fibrous cap thickness.	[98]
miR-146a and miR-181b	Polymeric Nanoparticles encapsulated in silicon microparticles	PEG/PEI complexes with miRNAs	Not specified	N/A	IV injection	15 μg of miRs in 100 μL, bi weekly for 12 weeks	ApoE−/− mice	Thioaptamer (ESTA) binding to E-selectin	∼60% and ∼50% decrease in lesion area in aorta compared to the control group when treated with NPs containing miR-146a and miR-181b respectively	[101]
miR-146	Superparamagnetic iron oxide nanoparticle (SPION)	Iron oxide	72.7 nm/−21.8 mV	275 strands per SPION	IV injection	10 mg/kg of Fe and 1.5 mg/kg of miRNA, twice a week for 3 weeks	ApoE−/− mice	Scavenger receptor class A (SR-A) mediated cellular uptake	Less toxicity due to being a non cationic nanomaterial Downregulation of genes related to the NF-κB pathway ∼30% decreased lesion areas in the aorta and aortic roots of treatment mice compared to the model mice	[107]
CCR2 siRNA	Polymeric Nanoparticle	Dextran	13.3 nm	N/A	IV injection	0.5 mg/kg	ApoE−/− mice	N/A	Significant reduction in inflammatory gene expression at the inflammation site.	[99]
CCR2 siRNA	Lipid Nanoparticle	C12-200 lipid, DSPC, cholesterol, PEG-DMG	70–80 nm	LE = 95%	IV injection	0.5 mg/kg, twice a week for 3 weeks	ApoE −/− mice	N/A	82% decreased macrophage number in atherosclerotic plaque in treatment compared to control	[102]
*Anti*-miR-712	Lipid Nanoparticle	DOTAP, DSPE-PEG2k, HSPC and cholesterol	167 ± 40 nm 44 ± 8 mV	LE = 95%	IV injection	1 mg/kg of anti miR-712 in 150 μL, injected at day 0, 3, 7, 10 and 14	ApoE−/− mice	VCAM-1 targeting peptide (VHPKQHR) binding to VCAM-1	Treatment group had a ∼66% reduced plaque size compared to the treatment group	[103]
*Anti*-miR33	Core-shell nanoparticle	Core: acetylated α−cyclodextrin Shell: PEG chains decorated with targeting moieties	147.5 ± 2.1 nm 9.9 ± 0.1 mV	LE = 88.1 ± 2.2%	IV injection	2 mg/kg, 2 injections first week followed by weekly injections for 2 months	ApoE−/− mice	cRGDfK peptide binding to αvβ3 integrin	Reduced macrophage population and matrix metalloproteinase-9 expression in plaques ∼64% reduction in total plaque area in treatment compared to the model	[105]
VE-cadherin siRNA	Liposome	POPC, SAINT-C18, Cholesterol, DSPE-PEG2000, and DSPE-PEG2000-Mal	106 ± 48 nm 3.8 ± 5 mV	LE = 71% ± 15	IV injection	10 μmol of total lipids/kg containing siRNA	HUVEC and HAEC for specificity and efficacy Male C57bl/6OlaHsd mice for PK	*anti*-E-selectin and *anti*-VCAM-1 antibodies binding to E-selectin and VCAM-1	Liposomes with *anti*-VCAM-1 antibodies showed increase in uptake of siRNA by HUVEC and HAEC compared to the *anti*-E-Selectin antibodies Downregulation of VE-cadherin mRNA by 60% and protein expression by 50% (HUVEC) and 25% (HAEC)	[100]
mTOR siRNA	Cerium oxide nanowire	Cerium oxide	146.4 ± 10.8 nm −12 ± 0.5 mV	LE = 70.6%	IV injection	0.5 mg/kg of siRNA	ApoE−/− mice	Stabilin-2-specific peptide (S2P) (CRTLTVRKC) binding to Stabilin-2	Higher uptake of S2P-PEGylated CeO_2_-NW in vitro and greater silencing of the mTOR gene compared to the CeO_2_-NW. *In vivo* studies showed comparable blood half life of ∼9.3 h The S2P- CeO_2_-NW showed a marked decrease in the progression of atherogenesis by a decrease in the atherosclerotic lesions by 67.3% compared to rapamycin (blocks mTOR) which showed a decrease of 41.3%	[106]
*Anti*-miR-712 (with carrier DNA having a complementary seq)	Gold nanospheres	Gold nanoparticle	5, 10, 20, 50 nm	N/A	–	–	immortalized mouse aortic endothelial cells (iMAECs)	VCAM-1-binding peptide (VHSPNKKGGSKGC) binding to VCAM-1	Best AuNP accumulation rate in left carotid artery was for particle size of 5 nm and showed specific delivery of *anti*-miR712 into VCAM-1 overexpressing cells	[104]

Several nanomaterials have been employed to deliver RNA therapies such as polymeric, lipid and metal oxide nanoparticles [[98], [99], [100], [101],[103], [104], [105], [106]]. Different polymeric nanoparticles such as PEG/PEI [101], dextran [99] and 7C1 [98] have been used as a delivery vehicle for various RNA therapies however their loading capacities and loading efficiencies weren't reported. Another limitation is the lack of in vitro release profiles of the NPs which isn't reported, making it difficult to compare different nanomaterials for the delivery of these RNA therapies.

**Fig. 5:**
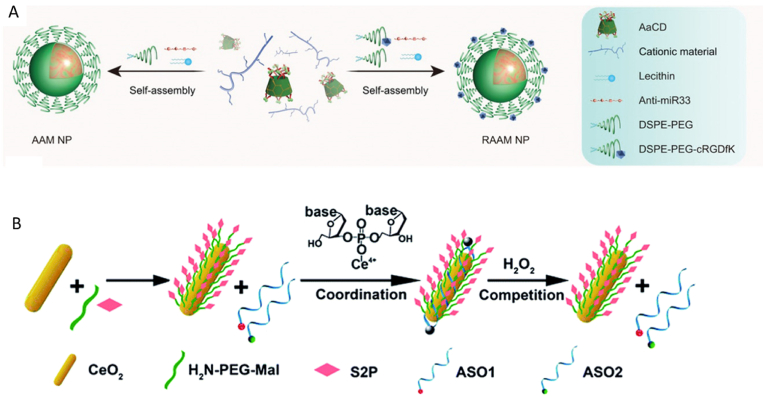
Different nanomaterial strategies employed to deliver nucleic acid therapies to the atherosclerotic plaque. **(A)** Cyclodextrin derived pH responsive nanoparticles targeting the integrin to deliver an ASO against *anti*-miR33 [105]. **(B)** H2O2-responsive and plaque-penetrating CeO2 NW. ASO1 and ASO2 are mTOR-specific sequences [106]. (ASO, antisense oligonucleotide; AAM, a pH-responsive *anti*-miR33 nanotherapy; RAAM, a pH-responsive cRGDfK-targeting *anti*-miR33 nanotherapy. Figures were reproduced with permission from Li et al., 2020 and Gao et al., 2018.

Li et al. [105] developed cyclodextrin derived pH responsive nanoparticles targeting the αvβ3 integrin to deliver an ASO against anti-micro-RNA-33 (miR33) (Fig. 5A). Micro RNA-33 is a key regulator of atherogenesis where an overexpression of miR33 in macrophages and hepatocytes leads to a decrease in cholesterol efflux, therefore, the inhibition of miR33 could have atheroprotective effects [108]. The pH-responsive NP consists of an acetylated cyclodextrin core, and a cationic material to enhance loading capacity and transfection efficiency. The shell of the NP was PEG chains decorated with a targeting peptide. As atherosclerotic lesions are acidic, the acid labile NPs can hydrolyse to release the *anti*-miR33 to the cells in the plaque. This strategy achieved a loading efficiency of 88.1 ± 2.2% with its in vitro release profile showing rapid release of the *anti*-miR33 at pH 5 with almost all the drug released (∼96%) in 60 h compared to a much slower release at pH 7.4 (∼40% cumulative drug release at 60 h). This study showed significant therapeutic effects in terms of plaque regression where a ∼64% reduction was observed in the total plaque area, a reduction in macrophage population and MMP-9 expression in the plaque in the treatment group compared to the model group.

Lipid nanoparticles are another type of drug delivery vehicle, where Kheirolomoom et al. [103] encapsulated *anti*-miR-712 with an efficiency of 95% in cationic lipoparticles. This particle was decorated with a vascular cell adhesion molecule 1 (VCAM-1) binding peptide for targeting. Mirco RNA-712 (miR-712), which is a mechanosensitive miRNA that is upregulated by disturbed flow (d-flow) in the endothelial cells, causes a decrease in expression of tissue inhibitor of metalloproteinase-3 (TIMP3), creating a cascade of events leading to pro atherogenic responses such as endothelial dysfunction and permeability. To increase the transfection efficiency of the miRNA, cationic lipid structures are used. However, exposed cationic membranes could lead to increased systemic toxicity therefore the cationic lipoparticles used in this study have the *anti*-miR complexed with the cationic lipid and coated with a neutral lipid [103]. The antiatherogenic activity of this therapy was performed on the partial carotid ligation model of ApoE−/− mice which showed a significantly reduced lesion area ∼43–45% lower compared to the untargeted NP and the NP containing a mismatched miR. Furthermore, no discernible offsite effects and toxicity were observed. Kowalski et al. [100] used a formulation of liposomes and a cationic amphiphile SAINT-C18 (1-methyl-4-(*cis*-9-dioleyl)methyl-pyridinium-chloride) labelled as SAINT-O-Somes to encapsulate siRNA against the endothelial gene vascular endothelial (VE) cadherin. This NP strategy was aimed at targeting inflamed endothelial cells on their role in the pathology of inflammatory diseases. These SAINT-O-Somes had a loading efficiency of 71 ± 15% and showed superior intracellular RNA release in endothelial cells due to its selectivity from the surface modification with VCAM-1 or E-selectin antibodies [100]. The study showed significant downregulation (up to 60%) of the target gene in vitro without affecting the viability of the cells.

Metal nanoparticles have also been used as carriers to deliver RNA therapies to the atherosclerotic plaque. Sun et al. [104] prepared gold nanospheres with its surface conjugated by *anti*-miR-712 and VCAM-1 binding peptide for the specific accumulation in the atherosclerotic plaque. Gold nanoparticles (AuNPs) are biologically inert, nontoxic and are easily synthesised, however, the main limitation of this strategy is the small size of the NP (5–50 nm) paired with its spherical shape making these particles prone to rapid clearance. Other inorganic NPs used to deliver RNA therapies include cerium oxide nanowire (CeO_2_ NW) where Gao et al. [106] created a nanoplatform of CeO_2_ NW functionalised with PEG and a stabilin-2 specific peptide ligand (S2P) where the CeO_2_ facilitates endosomal escape, PEGylation extends *in vivo* circulation time and the S2P for the targeted delivery of the siRNA for the mammalian target of rapamycin (mTOR) (Fig. 5B). This strategy had a loading efficiency of 70.6% and the drug delivery will be stimuli dependent as the Ce^4+^ binds more strongly to the O_2_^2−^ in H_2_O_2_ thereby taking advantage of the high H_2_O_2_ concentration in the plaque for the release of the siRNA. This is reflected in their *in vivo* studies which showed a significant decrease in the percentage of lesion area with the nanoparticle mediated therapy (∼5% lesion area of the whole aorta) compared with rapamycin (∼9% of lesion area of the whole aorta) and the model group which contained ∼16% of lesion area [106].

A recent study by Bai et al. [107], used PEG coated superparamagnetic iron oxide nanoparticles (SPIONs) with phosphorothioate (PS)-modified microRNA-146a attached to the PEG. MicroRNA 146a (miR-146a) is a key inhibitor to the proinflammatory NF-κB signalling pathway in macrophages and endothelial cells. Unlike liposomes and lipid nanoparticles, these SPIONS do not require any cationic or lipophilic transfection reagents as they are readily taken up by class A scavenger receptors (SR-A) present on cells. This was reported in a previous study where DNA oligos attached to PEG-SPIONs were readily taken up by SR-A in cells [109]. The miR146a is protected by nuclease degradation by the PS backbone, adding to the efficacy of the particle. The size and zeta potential of the particle was ∼72 nm and −21.8 mV. The *in vivo* studies showed reduced expression of genes involved in the NF-κB signalling pathway from the RNA of the aortas in the treatment mice compared to the control group. Furthermore, ∼30% less lesion area was observed in the treatment group compared to the control group. Despite these results, the blood circulation half-life of these nanoparticles was reported to be ∼2 h and the biodistribution was mostly reported in the liver (20–25%) and spleen (10–15%), and the aorta and the heart having only 1.2% of the injected nanoparticles.

These studies show how powerful RNA therapies are at silencing key factors for the development of atherosclerosis once their main drawbacks are addressed by nanoparticle mediated delivery. These include extending the circulation time of the RNA in the body by PEGylation of the NPs, adding site-specific ligands for specificity, and stimuli dependant mechanisms to ensure drug release at the intended site to minimise off-target effects. According to the current literature, the ideal nanoparticle strategy for the delivery of RNA therapies are liposomes and lipids as they hold the possibility of surface modifications to bind target specific ligands, in addition to the great loading efficiencies due to the aqueous core enabling the loading of this hydrophilic material. SPIONs can be considered as an ideal delivery vehicle as loading the decorated SPIONs do not require cationic agents to enter cells, and there was no report on severe systemic toxicity [107].

### 2.3 Strategies for the targeted delivery of anti-inflammatory/chemotherapeutic drugs

Several anti-inflammatory therapies have been conducted in clinical trials in an attempt to treat atherosclerosis such as the CANTOS [19], CIRT [20] and LoDoCo [21] studies. Methotrexate (MTX) which is a chemotherapeutic drug successfully used to treat chronic inflammatory diseases such as rheumatoid arthritis is also being tested for its atheroprotective properties. This is because, at high doses, MTX is a cytostatic drug however, at low doses, it exhibits anti-inflammatory properties. Table 3 provides a summary of the nanoparticle mediated delivery of anti-inflammatory and chemotherapeutic drugs to the atherosclerotic plaque.

**Table 3:** Nanoparticle strategies for the delivery of ani inflammatory/chemotherapeutics to the atherosclerotic plaque.

Therapeutic Agent	Nanoparticle	Key nanoparticle materials	Size/Charge	Loading capacity (LC) and loading efficiency (LE)	Route of delivery	Injected dose	Model	Targeting strategy	Key Findings	Reference
**Methotrexate (MTX)**	Polymeric Nanoparticle	PLGA core stabilised with EGG-PG and DSPE-PEG	100 nm −60 mV	LE = 57.4% LC = 2.8%	IV injection	20 μg of MTX, bi weekly for 4 weeks	ApoE−/− mice	N/A	Reduced plaque burden by 50% in treatment group compared to control group, in high fat diet mice in 1 month.	[110]
**Dexamethasone**	Liposome	DPPC and PEG-2000-DSPE	100 nm; 5.1±1.4 mV	LC = 0.13 mg dexamethasone phosphate per μmol of phospholipid	–	–	*In vitro* model using peripheral blood mononuclear cells (PBMC)	N/A	Inhibited migration of monocytes Reduced release of TNFa and IL6 which are proinflammatory cytokines.	[111]
**Didodecyl-methotrexate (ddMTX)**	Lipid core nanoparticles (LDE)	Lipid mixture (Cholesterol oleate, phosphatidylcholine, triolein and cholesterol)	<220 nm	LC = 0.2 mg/mg NP	IV injection	4 mg/kg of MTX, injected once per week for 4 weeks	Male NZ white rabbits. 1% cholesterol diet- 8wks	LDE is taken up by the cells through the LDL receptor mediated endocytic pathway	65% lesion reduction in LDE-ddMTX compared to control Decrease in gene expression of TNF-α, VCAM-1 IL-1β, MCP-1, IL-18, MMP-9 and MMP-12 in aortic arch of treatment group.	[112]
**Paclitaxel**	Lipid core nanoparticles (LDE)	Lipid mixture (Cholesterol oleate, phosphatidylcholine, miglyol and cholesterol)	N/A	LC = 60 mg of PTX were added in 606 mg of lipids	Intraperitoneal injection	4 mg/mL, weekly for 4 weeks	LDLR −/− mice	LDE uptake by different mechanism and not through the LDL receptor pathway	Reduction of lesions in wall area (14%) and stenosis (22%) observed by MRI in treatment compared to model	[113]
**Paclitaxel**	Lipid core nanoparticles (LDE)	Lipid mixture (Cholesterol oleate, phosphatidylcholine, miglyol and cholesterol)	40.69 ± 1.44 nm and 83.61 ± 1.85 nm	LC = 60 mg of PTX were added in 606 mg of lipids	IV injection	4 mg/kg of PTX, injected once per week for 4 weeks	Male NZ white rabbits. 1% cholesterol diet- 8wks	LDE is taken up by the cells through the LDL receptor mediated endocytic pathway	Size of the NPs do not significantly impact the treatment 54–56% decrease in the lesion area of the rabbit aortas from the treatment compared with the control	[114]
**Paclitaxel oleate (PTX) and didodecyl methotrexate (MTX)**	Lipid core nanoparticles (LDE)	Lipid mixture (Cholesterol oleate, phosphatidylcholine, triglycerides and cholesterol)	LDE-PTX and LDE-MTX = 45–60 nm	LC = 60 mg of PTX or MTX were added in 322 mg of lipids	IV injection	4 mg/kg of PTX and MTX, injected once per week for 4 weeks	Male NZ white rabbits. 1% cholesterol diet- 8wks	LDE is taken up by the cells through the LDL receptor mediated endocytic pathway	The combination therapy of LDE-PTX + LDE-MTX shows decreased lesion area of the total artery (19%) compared to the control (63%)	[115]
**Docetaxel (DTX) (derivatized to enhance lipophilicity)**	Lipid core nanoparticles (LDE)	Lipid mixture (Phosphatidylcholine, esterified cholesterol, non-esterified cholesterol and triglycerides)	60 nm	1:10 ratio of DTX to LDE	IV injection	1 mg/kg once a week for 4 weeks	Male NZ white rabbits. 1% cholesterol diet- 8wks	LDE is taken up by the cells through the LDL receptor mediated endocytic pathway	LDE-DTX showed 80% less atherosclerotic area vs. LDE control The microscopic lesions and VSMCs in intima were 85% lower in LDE-DTX vs. LDE control.	[116]
**Rosiglitazone (Rosi)**	Lipid latex (LiLa) nanoparticles	PtdSer, 9-CCN, and phosphatidylethanolamine-PEG2000	65 ± 10 nm	LC (mg/1 mg latex); Rosi = 0.59 ± 0.15; TAM = 0.48 ± 0.39; PAX = 0.14 ± 0.07	No *in vivo* study	–	*In vitro* model using RAW 264.7 macrophages	Phosphatidylserine (PtdSer) and oxidised cholesterol ester derivative cholesterol-9-carboxynonanoate (9-CCN) which promote phagocytosis by macrophages	Rosi-LiLa model displayed a higher loading capacity Preferential uptake of LiLa NPs in RAW cells Free Rosi and Rosi-LiLa showed significant decrease in the concentration of proinflammatory cytokines.	[117]
**Paclitaxel (PTX)**	Mesoporous silica nanomotor	Fe_3_O_4_ nanoparticles, amine modified mesoporous silica nanoparticles	450 nm; −11.85 mV	20 mg of PTX in 20 mg of amine-modified mesoporous silica	Direct delivery by drug-coated balloons	20 mg of PTX	NZ white rabbits	Anti VCAM-1 polyclonal antibody binding to VCAM-1	The plaque area of the nanomotor treatment group was smaller compared to the PTX only control group No damage was observed in the blood vessels due to photothermal treatment	[118]

**Fig. 6:**
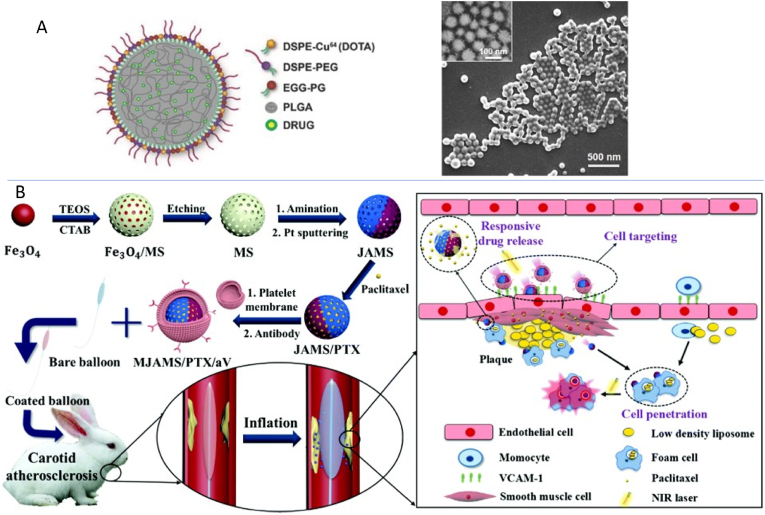
Delivery of anti-inflammatory drugs to the atherosclerotic plaque using different nanomaterial strategies. **(A)** Schematic illustration of spherical polymeric nanoconstructs (SPN) with scanning electron microscopy image of the MTX-SPNs [110]. **(B)** Schematic illustration of the synthesis of the nanomotor and the treatment mechanism of the nanomaterial coated balloon [118]. MS, mesoporous silica; JAMS, Janus aminated mesoporous silica; PTS, Paclitaxel; aV, anti VCAM-1 antibody. Figures were reproduced with permissions from Stigliano et al., 2017 and Huang et al., 2020.

As MTX is a lipophilic drug, it has been loaded in polymeric [110] and in lipid core particles [112,115]. Stigliano et al. [110] loaded MTX into spherical polymeric nanoconstructs (SPN) which contained a hydrophobic PLGA core stabilised by phospholipids and PEG chains where radioactive ^64^Cu and fluorescent molecules were conjugated to the phospholipid monolayer for imaging (Fig. 6A). As spherical shapes showed greater uptake by immune cells [45], the MTX was released into the cell after the endosomal digestion of the SPN. This strategy had a loading efficiency of 57.4% and the in vitro release showed ∼90% of the MTX being released within the first 24 h. The atheroprotective effect of this therapy showed a decrease in plaque burden in the ApoE−/− mice in the MTX SPNs by 50% compared with the control and MTX only.

Bulgarelli et al. [112] used a lipophilic derivative of MTX, didodecyl methotrexate (ddMTX) to be loaded into a cholesterol rich nanoemulsion (LDE). These LDE-ddMTX showed greater cytotoxicity with reduced haematological toxicity [112]. The *in vivo* results of atherosclerotic rabbits showed 65% less lesions in the total aorta in the treatment group compared to the control group. Since LDE resembles the structure of LDL molecules, they are taken up by the LDL receptors (LDLR) or LDL receptor related proteins (LRP-1). This was shown by a study conducted by Lima et al. [113], where they used LDLR knockout mice to determine if the LDE NPs were taken up primariliy by the LDLR pathway. Paclitaxel (PTX), a chemotherapeutic drug which acts as an anti-proliferative, was loaded into the LDE NPs which showed improved therapeutic efficacy against the model group where a reduction in the aortic lesion area and reduced stenosis was observed in the treatment group.

Freitas et al. [114] synthesised small particles (40.69 ± 1.44 nm) and large particles (83.61 ± 1.85 nm) of LDE NPs, loaded with PTX to determine if the size of the NP affected the treatment outcome in atherosclerotic rabbits. This study showed that size plays no significant role in the cellular uptake of the LDE particles with both particles showing similar regression in the atherosclerotic plaque. In order to determine if the combination therapy of LDE-MTX with LDE-PTX could accelerate the regression of atherosclerosis compared with LDE-PTX alone, Gomes et al. [115] conducted an animal study where the atherosclerotic rabbits showed both the treatment methods increase the regression of the plaques with the combination group having a 10% more increase in the regression of the plaque (49% reduction in plaque area in total aorta area for LDE-PTX compared to 59% reduction for LDE-PTX + LDE-MTX). Both therapies show comparable anti-inflammatory activity with decreases in gene expression of tumor necrosis factor alpha (TNFα) (65% and 79% reduction in LDE-PTX and PDE-PTX + LDE-MTX respectively) and expression of MMP9 (74% and 78% reduction in LDE-PTX and PDE-PTX + LDE-MTX respectively). Overall, this study showed that the use of combined therapy produces stronger effects on the regression of the atherosclerotic plaque. Paclitaxel was further used in a novel approach by Huang et al. [118]. Different to the other studies in this review which focuses on introducing nanomedicine to the circulation, this study employed another strategy that involves drug loaded nanomaterial coating on a balloon catheter device (Fig. 6B). The nanomotor was produced by aminating mesoporous silica particles, with one side of the particle sputtered with platinum (Pt) and then PTX and an *anti*-VCAM-1 antibody was modified onto the nanomotor before covering it with a platelet membrane to avoid leakage of drugs. Once the catheter is inserted, the nanomotors will bind to the VCAM-1 expressing plaque and near infrared (NIR) irradiation acts as the driving force by stimulating Pt to penetrate the plaque for releasing the drugs. This study showed good biocompatibility and no damage was observed in the blood vessels of the animals due to this photothermal treatment. Other anti-inflammatory drugs such as dexamethasone [111] were encapsulated in liposomes showing the induction of pro-inflammatory cytokines thereby inhibiting monocyte and macrophage recruitment in *in vitro* studies.

Meneghini et al. [116] also used LDE nanoparticles as a vehicle for docetaxel (DTX), which is another taxane such as paclitaxel, to determine its anti-atherosclerotic activity. The animal studies involving atherosclerotic rabbits showed an 80% reduction of the atheroma area in LDE-DTX compared to LDE only control group.

Currently, the ideal strategy for the delivery of anti-inflammatory or chemotherapeutic agents are cholesterol rich nanoemulsions (LDE) which are lipid nanoparticles resembling LDL molecules. These particles have shown good drug loading associated with the hydrophobic core when synthesising as well as great uptake in the cells by the LDL receptor mediated endocytic pathway. Furthermore, no observable toxicity was reported in all the studies utilising LDE nanoparticles. A strong association of these chemotherapeutic drugs to LDE is observed, which significantly reduces the toxicity of the drug, possibly due to the lower doses required to achieve a therapeutic effect. Compared to polymeric NPs, the lipid nanoparticles showed greater loading capacities as well. In terms of *in vivo* efficacy of these treatments, similar results were observed with MTX, PTX and DTX using LDE NPs as the drug delivery system in NZ white rabbits. A comparison of the same drug with a different nanomaterial carrier cannot be made due to the difference in the animal models used. Another limitation is the lack of in vitro release data in most studies, thus it is challenging to compare the different NPs used. However, there is a clinical trial ongoing with LDE-MTX at phase 2 and LDE-Placebo at phase 3 (ClinicalTrials.gov Identifier: NCT04616872) [119] indicating the translational potential of these nanomaterials. Although many studies haven't utilised polymeric nanoparticles for the delivery of these drug types to the atherosclerotic plaque, they too show potential due to the ability of surface modifications to avoid liver uptake compared to LDE nanoparticles.

Antibody-based therapeutic approaches for mitigating inflammation in atherosclerosis is another promising treatment path, which is evaluated in recent preclinical and clinical findings by Ji et al., 2021 [120]. Although showing positive results, exploring the use of nanomaterial-mediated delivery could further improve its clinical outcome. Inorganic NPs like gold, silica, or superparamagnetic nanoparticles can be utilised to deliver these antibody therapies, as the antibodies can be conjugated by adsorption or covalent bonding. Some inorganic NPs may be used as diagnostic markers for MRI (SPIONs) or CT (AuNPs) due to their unique physical properties that help with biomedical imaging [121]. Polymeric NPs or liposomes can also be used to deliver antibody therapies as they allow for the co-delivery of a different drugs encapsulated within the NP for a greater therapeutic effect.

### 2.4 Strategies for the targeted delivery of other small molecule drugs

Several small molecule drugs have used nanoparticles as drug delivery vectors to improve the specificity and efficacy of the drug. The majority of the small molecule drugs reviewed are hydrophobic employing polymeric nanoparticles for the delivery (Table 4).

**Table 4:** Nanoparticle strategies for the delivery of other small molecule drugs to the atherosclerotic plaque.

Therapeutic Agent	Nanoparticle	Key nanoparticle materials	Size/Charge	Loading capacity (LC) and loading efficiency (LE)	Route of delivery	Injected dose	Model	Targeting strategy	Key Findings	Reference
**CCR2 antagonist (Teijin compound 1)**	Target sensitive liposome (TSL)	DOPE, DOPA and Mal-PEG-DSPE	128 ± 19 nm	N/A	–	N/A	ApoE−/− mice aortas used for in situ study	VCAM-1 targeting peptde (VHPKQHRGGSKGC) binding to VCAM-1	*In vitro* tests of the target sensitive liposome coupled with a VCAM-1 peptide (Vp-TSL) binds to the cells expressing VCAM-1 and releases Teijin The targeted liposomes showed a 78% release of Teijin after 6 h compared to the ∼30% in the non-targeted liposomes The inhibition of monocyte/macrophage infiltration into the aorta of ApoE−/− mice were shown in situ.	[122]
**synthetic LXR agonist GW3965 (GW)**	Polymeric Nanoparticle	PLA, DSPE, mPEG, DLPC	83.9 ± 2.6 nm; 1.8 ± 0.8 mV	LE: 45%	IV injection	8 mg/kg, twice a week for 5 weeks	LDLR−/− mice	Collagen IV binding peptide (KLWVLPKGGGC) targeting Collagen IV	Decrease in plaque macrophages by ∼30% compared with free drug Hepatic triglyceride and cholesterol were lower on Col (IV) GW-NPs compared to PBS whereas free GW showed increased levels	[123]
**LXR agonist GW3965**	Polymeric Nanoparticle	PLGA-b-PEG	156.6 ± 10.3 nm	LE = 58.8 ± 1.3% LC = 9.8 ± 0.2%	RO injections	10 mg/kg, 3 times a week for 2 weeks	LDLR−/− mice	Phosphatidylserine (PS) lipid which promote phagocytosis by macrophages	Reduction in the CD68-positive macrophage content in lesions by 50% No increase in triglycerides or total cholesterol in the plasma and liver Decreased the inflammation and increased the LXR gene expression compared to free GW3965.	[124]
**SRT1720**	Mesoporous silica Nanoparticle	Silica	61.4 ± 7.9 nm; 8.6 ± 0.3 mV	LC = 47 ± 4%; LE = 42 ± 2%	Intraperitoneal injection	170 mg/kg per day, every other day for 4 weeks	ApoE−/− mice RAW264.7 mouse cells were used for in vitro	AntiCD36 antibody targeting the CD36 receptor expressed on the surface of macrophages	Decrease in the blood total cholesterol was observed in vitro with inhibition of macrophage foaming A significant improvement in the serum total cholesterol and aortic plaque Enhanced therapeutic efficacy of NP-SRT1720 compared to the free drug ∼32% decrease in plaque area of the whole aorta in the treatment group compared to the model	[125]
**Rapamycin**	Polymeric Nanoparticle	β-cyclodextrin (β-CD)	RAP/Ac-bCD NP (186.5 ± 1.6 nm, −34.3 ± 1.4 mV) RAP/Ox-bCD NP (253.5 ± 3.9 nm, −22.8 ± 0.5 mV)	LC: RAP/PLGA NP (8.0%); RAP/Ac-bCD NP (12.5%); RAP/Ox-bCD NP (7.0%)	Intraperitoneal injection	3 mg/kg every 3 days for 2 months	ApoE−/− mice	N/A	Both types of stimuli responsive therapies delayed the progression of atherosclerosis Enhanced the stability of atherosclerotic lesions Plaque area decreased from 30.2% (model group) to 5.1% (RAP/Ac-bCD) and 4.5% (RAP/Ox-bCD)	[126]
**Pioglitazone**	Polymeric Nanoparticle	PLGA	247 nm diameter	N/A	IV injection	7 mg/kg per week for 4 weeks	ApoE−/− mice	N/A	NP mediated delivery of pioglitazone in mouse model inhibited atherosclerotic plaque destabilisation and rupture The polarity of the macrophages was regulated to be less inflammatory No significant difference in the plaque area between treatment and control groups	[127]
**1,25-dihydroxyvitamin D3 (aVD) and ApoB-100-derived antigenic peptide P210**	Polymeric Nanoparticle	poly (ethylene glycol)-bl-poly (propylene sulfide) (PEG-b-PPS)	143.6 nm; −5.32 ± 1.24 mV	LC = 12.5 μg P210/100 ng aVD/1.5 mg polymer	IV injection	1 μg/ml of aVD in 100 μL, injected every week for 8 weeks	ApoE −/− mice	P-D2 peptide (GGVTLTYQFAAGPRDK) binding to the CD11c present on the surface of dendritic cells	Decreased atherosclerotic lesions and presence of macrophages in the P210/P-D2-PEG5-PS-aVD group compared to the control, free aVD and aVD loaded PS.	[128]
**SHP1i (a small-molecule inhibitor of CD47's downstream effector molecule)**	Carbon nanotube	Single walled carbon nanotubes	5–6 nm diameter, >60 nm length −7.19 ± 2.53 mV	N/A	IV injection	N/A	ApoE−/− mice	N/A	Promoted efferocytosis resulting in the reduced lesion area and necrotic core	[129]
**Superoxide dismutase mimetic agent (Tempol) and hydrogen peroxide-eliminating compound**	Polymeric Nanoparticle	β-cyclodextrin (β-CD)	128 ± 1 nm	N/A	IV injection	100 mg/kg of NP (17.2 mg/kg of Tempol) for 9 weeks	ApoE−/− mice	Passive targeting by EPR	Decreased necrotic core with thicker fibrous cap Plaque stabilised with fewer cholesterol crystals Decreased levels of macrophages and matrix metalloproteinase-9 levels Decreased average plaque area from 24.9% (control) to 6.3% (treatment group)	[130]

Poly D,L-lactic-co-glycolic acid (PLGA), is a popular polymeric nanomaterial used as a drug delivery vehicle for small molecule drugs, such as pioglitazone [127] and liver X receptor (LXR) agonist, GW3965 [123,124]. Poly (ethylene glycol) was used as a coating in most of the polymeric nanoparticles reported due it its crucial properties which improve the biophysical and chemical properties of the nanoparticle [131].

Yu et al. [123] and Zhang et al. [124] have both employed polymeric NPs to encapsulate liver X receptor (LXR) agonist, GW3965. The activation of LXR exerts several atheroprotective effects. However, this could also lead to the induction of the Sterol regulatory element-binding protein 1 (SREBP-1c), which causes hypertriglyceridemia and hepatic steatosis. By encapsulating the LXR agonists in polymeric NPs, these adverse effects were mitigated. Both studies showed comparable loading efficiencies (45% [123] and 58.8% [124]) of the drug and reduced the macrophage content in the lesion sites without increasing the total cholesterol in the liver and plasma triglycerides [123,124]. Zhang et al. [124] synthesised NPs through the self-assembly of a biodegradable diblock poly (lactide-co-glycolide)-b-poly (ethylene glycol) (PLGA-b-PEG) copolymer, while Yu et al. [123] used a biodegradable hydrophobic (poly (d,l-lactide) (PLA) core functionalised with a layer of methoxy (polyethylene glycol) (mPEG) and a collagen IV peptide for targeting. Both these studies however didn't mention the effect GW3965 had on the atherosclerotic plaque to make a comparison on the effect of the different polymers with or without targeting ligands.

Yi et al. [128] engineered polymersomes, which were synthesised using polyethylene glycol-block-polypropylene sulphide (PEG-b-PPS) with an ApoB-100 derived antigenic peptide P210 as a targeting ligand to encapsulate an anti-inflammatory agent 1, 25-Dihydroxyvitamin D3 (aVD). While aVD promotes the maintenance of immature tolerogenic dendritic cells (DCs), which inhibit pro-inflammatory T cells, P210 reduces atherosclerosis in hyperlipidemic animals. The NPs were also decorated with a P-D2 peptide which targets the CD11c on the surface of dendritic cells. Flow cytometric analysis showed the significantly higher uptake of the targeted NP in the atheroma in the mice than the untargeted NPs. This study also showed reduced lesion area (∼33% decrease) in mice treated with the aVD loaded polymersomes compared with the model and ∼20% decrease compared to the untargeted NP, inferring that targeted deliveries produce greater efficacy in the treatment of the plaque.

**Fig. 7:**
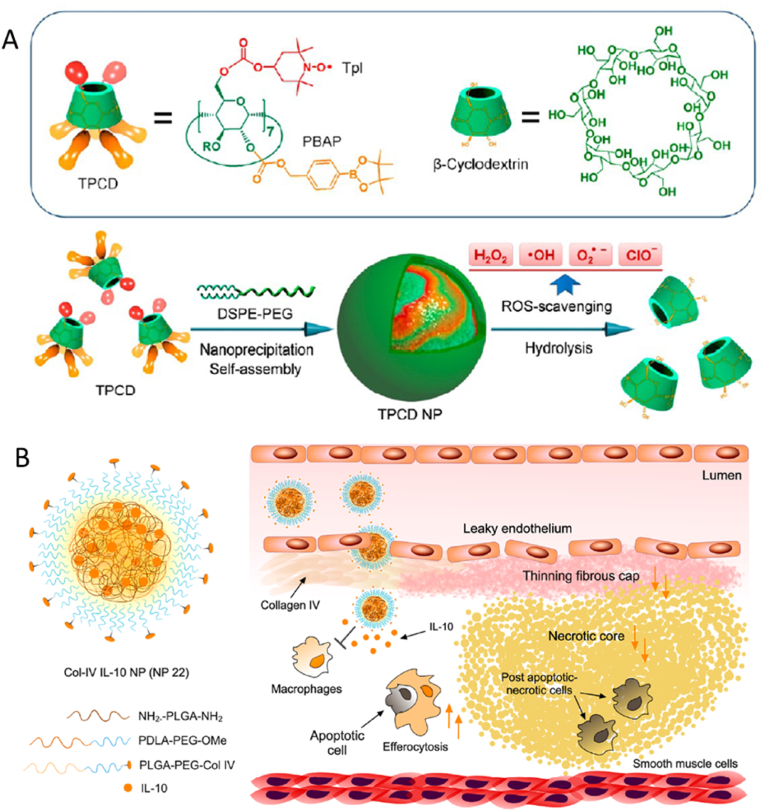
Different types of polymeric nanoparticles used to deliver small molecule drugs and peptides to the atherosclerotic plaque. **(A)** Schematic representation of the ROS scavenging nanoparticle [130]. **(B)** Collagen IV targeting NP design encapsulating cytokine IL-10, with the method of action displaying the release of IL-10 to the atherosclerotic site [133]. Tpl, tempol; PBAP, phenylboronic acid pinacol ester; IL-10, Interleukin-10. Figures were reproduced with permission from Wang et al., 2018 and Kamaly et al., 2016.

Beta cyclodextrin (bCD) is another polymer which is used for the delivery of small molecule drugs. Dou et al. [126] compared the non-stimuli responsive PLGA NP with a pH responsive acetylated β-cyclodextrin (bCD) (Ac-bCD) and a ROS-sensitive bCD material (Ox-bCD), loaded with rapamycin. Wang et al. [130] utilised β-cyclodextrin (bCD) to conjugate a small molecule, Tempol, which is a superoxide dismutase mimetic agent that scavenges oxygen free radicals and converts superoxide to oxygen and H_2_O_2_ [132], and a H_2_O_2_-eliminating compound of phenylboronic acid pinacol ester (Fig. 7A). Both these strategies showed similar regression of the atherosclerotic plaque area in the treatment groups compared to the model.

Other types of nanomaterials are mesoporous silica nanoparticles loaded with SRT1720 (a specific activator of SIRT1) [125]. SIRT1 has many atheroprotective effects such as mediating vasodilation, downregulating the expression of pro inflammatory cytokines, promoting reverse cholesterol transport, stabilising plaques, and preventing thrombosis. In this study, the NP was also conjugated with antiCD36 antibody to specifically bind to macrophages in the atherosclerotic plaque. Compared to the free SRT1720, this therapy showed enhanced therapeutic efficacy by improving the total cholesterol level and plaque area (∼15.2% plaque area of the whole aorta in the treatment group compared to ∼23.2% plaque area in the model group) [125].

The use of single walled carbon nanotubes (SWNT) to load a chemical inhibitor of the antiphagocytic CD47-SIRPα signalling axis have been studied by Flores et al. [129]. CD47 is a ligand that binds to the signal regulatory protein α (SIRPα) on macrophages, which in turn activates the Scr homology 2 domain containing phosphatase-1 (SHP-1) causing a signalling cascade suppressing phagocytic function, preventing efferocytosis and promoting plaque expansion. This study prevents this signalling pathway by loading a small molecule inhibitor of SHP-1 into the SWNTs and functionalising it with PEG to enhance the loading capacity and minimise toxicity. The *in vivo* studies showed accumulation in atherosclerotic lesions and uptake by lesional macrophages showing significant anti atherosclerotic effect [129]. These functionalised SWNTs showed no acute or chronic toxicities to the mice and showed no association to anaemia which is a major adverse effect of pro-efferocytotic antibody therapy.

Despite having multiple nanomaterials to deliver small molecules to the atherosclerotic plaque, the most suitable strategy for hydrophobic drugs are polymeric nanoparticles. There are different polymers, each with unique characteristics which are suitable for different types of drugs. These allow for the synthesis of particles with controllable size, good loading efficiencies and the option for surface modifications to enhance the efficacy of the treatment. For example, Dou et al. [126] and Wang et al. [130], showed superior efficacy of bCD with its targeted delivery and stimuli response release of rapamycin and tempol, respectively. Yi et al. [128] and Yu et al. [123] attached targeting ligands to their polymeric NPs and showed the increased therapeutic efficacy of their treatment.

### 2.5 Strategies for the targeted delivery of peptides

Not many studies have been conducted on the use of peptides as a therapy for atherosclerosis (Table 5). Due to their small size, peptides are rapidly cleared from plasma therefore requires repeated administrations of large doses for an efficacious treatment. A couple of studies conducted by Fredman et al. [134] and Kamaly et al. [133] utilised PLGA-PEG NPs which were decorated with collagen IV binding peptides for targeting, to deliver an Annexin A1 mimetic peptide (Ac2-26) [134] and Interleukin-10 (IL-10) (Fig. 7B) [133]. Both Ac2-26, which is an annexin A1 mimetic peptide that mediates inflammation resolution and provides protective actions by the activation of N-formyl peptide receptor 2 (FPR2/ALX), and IL-10, which is an anti-inflammatory cytokine, provide anti-atherosclerotic behaviour. These NPs showed a comparable loading capacity of 4% (w/w) of Ac2-26 and 1.75–2.62% (w/w) of IL-10. The *in vivo* results of both studies show decrease in lesion area and necrotic core in the treatment compared to the non-treatment group.

**Table 5:** Nanoparticle strategies for the delivery of peptides to the atherosclerotic plaque.

Therapeutic Agent	Nanoparticle	Key nanoparticle materials	Size/Charge	Loading capacity (LC) and loading efficiency (LE)	Route of delivery	Injected dose	Model	Targeting strategy	Key Findings	Reference
IL-10	Polymeric Nanoparticle	PLGA-PEG	between 76.23 ± 1.1 nm and 212.66 ± 9.5 nm; −32 to −8 mV	LE = between 65.57% and 98.19% LC = between 1.75% and 2.62% (w/w % of polymer mass)	IV injection	5 μg of IL-10 per injection	Ldlr^−/−^ mice	Collagen IV binding peptide (CGGGKPLVWLK) targeting collagen IV	Decreased necrotic core Increased lesional efferocytosis Thicker fibrous cap	[133]
Annexin A1 mimetic peptide (Ac2-26)	Polymeric Nanoparticle	PLGA-PEG	<100 nm < −30 mV	LC = 4% Ac2-26 (w/w)	IV injection	10 μg of peptide per injection	Ldlr^−/−^ mice	Collagen IV binding peptide (CGGGKPLVWLK) targeting collagen IV; Ac2-26 peptide (AMVSEFLKQAWFIENEEQEYVQTVK) binds to the N-formyl peptide receptor 2 (FPR2/ALX) expressed on macrophages	Selective binding to the plaque in Col IV–Ac2-26 NPs compared with NPs without Col IV binding peptide Significant decrease in plaque area and necrotic core in the treatment group vs the control group Increased fibrous cap thickness stabilising plaque preventing likelihood of rupture	[134]

As mentioned previously, most peptides are used as targeting ligands to the atherosclerotic plaque with only a couple of studies utilising peptides for the treatment of atherosclerosis. Despite having limited information for comparison of nanomaterials, polymeric NPs are the ideal choice for delivering peptides due to its versatility.

## 3 Conclusions and perspectives

Atherosclerosis is a chronic inflammatory disease which is the main cause for cardiovascular diseases which account for millions of deaths each year [1]. There are several clinical therapies that exist to control the plaque formation by managing the risk factors or invasive surgical procedures. As there are so many types of drugs used for the treatment of atherosclerosis, there is also an increasing interest in nanoparticles for the targeted delivery of drugs to the atherosclerotic plaque. This review discussed the efficiency and efficacy of current drugs used to treat atherosclerosis employing nanomaterials for their delivery. The nanomaterial used depends on the type of the drug used and its solubility. We have reviewed the most popular drug types: (i) statins, (ii) nucleic acid therapies, (iii) anti-inflammatory/chemotherapeutic agents, (iv) other small molecule drugs and (v) peptides. The ideal nanomaterial used for these particular drug types is inferred despite the limitations present in the literature.

Several nanomaterials were discussed with the most common type of nanomaterial being lipid nanoparticles followed by metal and polymeric nanoparticles (Fig. 1). Each nanomaterial possesses distinct characteristics. As it is challenging to compare treatment efficacy of different delivery systems for a specific type of drugs due to limited information presented in the literature and differences in experiment settings and nanoparticle doses used, the selection of an optimal nanomaterial is contingent upon payload. Lipid nanoparticles are ideal carriers due to their synthesis from biocompatible and biodegradable building blocks. They can encapsulate both hydrophobic (within the lipid bilayer) and hydrophilic drugs (in the aqueous core). Despite this, balancing the drug loading capacity and maintaining an optimal particle size presents a significant challenge with these particles. Other disadvantages of these NPs include lack of targeting ability and short blood circulation which can be addressed by modifying the NP. Polymeric NPs can incorporate both hydrophobic and hydrophilic drugs as well with the polymers having tuneable chemical and physical properties that can release the payload in a sustained manner or in a stimuli responsive manner. Like lipid NPs, these particles can load both hydrophobic and hydrophilic drugs, albeit the degree of encapsulation depends on the molecular weight of the polymers [135]. Metal nanoparticles can be functionalised with peptides, nucleic acids and even targeting ligands. However, their application is often limited by challenges related to stability and toxicity. Aside from the nanomaterials discussed, DNA nanostructures are another attractive platform that could be used for drug delivery [136]. Currently, no study has been conducted to assess DNA nanostructures to deliver anti atherosclerotic therapies. Advantages of using DNA nanostructures include the ease of synthesis where the size and shape can be controlled as well as the precise decoration of molecules in the structure. Despite this, major challenges such as the stability of the particles and endosomal escape need to be overcome. This is discussed extensively in a review by Lacroix et al., 2021 [137]. Atherosclerosis is a chronic disease. The longer it progresses, the more difficult to treat. It is advisable to take measures to prevent the formation of atherosclerosis. Despite several nanoparticle mediated therapies showing promising results in preliminary studies, there are still obstacles that need to be addressed. Nanotoxicology which includes the biocompatibility, pharmacokinetics and the biosafety of the nanomaterials should be evaluated in animal models [138]. Although mice represent the model of choice for initial testing and safety, larger animal models such as pigs and non-human primates represent an optimal model due to their similarity in physiology, metabolism, and cardiovascular anatomy [139]. The differences in the preclinical animal models with humans also presents an issue when determining the safety between species [139]. The scale up and production under good manufacturing process (GMP) regulation is another hurdle that needs to be overcome for the approval of novel nanomaterial mediated therapies for clinical use. This involves a robust manufacturing workflow involving quality control to ensure that the properties aren't altered during the scale-up [139]. At the time of writing, only a very small percentage of nanomaterials in clinical studies are directed for the treatment or diagnosis of atherosclerosis or any other cardiovascular disease [119]. With the escalating demand for innovative nanomaterial strategies in addressing atherosclerosis, it is important to consider the various challenges faced when translating from a proof-of-concept to clinical investigations.

## Declaration of competing interest

The authors declare that they have no known competing financial interests or personal relationships that could have appeared to influence the work reported in this paper.

## Data Availability

No data was used for the research described in the article.

## List of Abbreviations

ASO: - antisense oligonucleotides
bCD: - beta cyclodextrin
CD: - cyclodextrin
CVD: - cardiovascular diseases
IV: - Intravenous
EC: - endothelial cells
HA: - hyaluronic acid
HDL: - high density lipoprotein
ICAM: - intracellular adhesion molecule
LDE: - cholesterol rich nanoemulsion
LDL: - low-density lipoprotein
miRNA: micro-RNA
MMP: matrix metalloproteinase
NP: nanoparticle
ox-LDL: oxidised low-density lipoprotein
PEG: polyethylene glycol
PLGA: poly(lactic co-glycolic acid)
ROS: reactive oxygen species
siRNA: small interfering RNA
VCAM: vascular cell adhesion molecule
VSMCs: vascular smooth muscle cells
